# Effects of a Real Exposure Class XC4 Mediterranean Climate Environment in the Behavior of Mortars Made Using Ternary Binders with Addition of Slag, Fly Ash and Limestone

**DOI:** 10.3390/ma14195848

**Published:** 2021-10-06

**Authors:** Javier Ibáñez-Gosálvez, Teresa Real-Herraiz, José Marcos Ortega

**Affiliations:** 1 Departamento de Ingeniería Civil, Universidad de Alicante, Ap. Correos 99, 03080 Alicante, Spain; javier.ibanez@ua.es; 2 Instituto de Matemática Multidisciplinar, Universidad Politécnica de Valencia, Camino de Vera s/n, 46022 Valencia, Spain; tereaher@upv.es

**Keywords:** ternary binders, Mediterranean climate environment, real condition exposure, class XC4, fly ash, ground granulated blast-furnace slag, limestone, mechanical properties, microstructure, durability

## Abstract

For improving the contribution of the cement industry to mitigate global warming, many strategies have been put into practice, such as the use of eco-friendly cements with the incorporation of additions substituting clinker. Nevertheless, the use of ternary binders for the production of commercial cements is still reduced, particularly in Spain. The purpose of this research is to characterize the long-term influence produced by the exposure to a real in situ inland Mediterranean climate condition in the pore network, parameters related to durability and mechanical performance of mortars made with ternary binders, which incorporated limestone, fly ash, and ground granulated blast-furnace slag, in comparison with mortars without additions and binary blended mortars. The site verified the specifications of exposure class XC4 of Eurocode 2. The ternary and binary binders accomplished the prescriptions of cement type CEM II/B. The pore network was studied with mercury intrusion porosimetry and electrical resistivity. Water absorption, diffusion coefficient, carbonation depth, ultrasonic pulse velocity, compressive and flexural strengths have been determined. The exposure to the environment produced after 250 days an increase in porosity, a loss of pore refinement, a rise of the carbonation depths, and a reduction in the mechanical strengths, highlighting the better overall performance of ternary mortar with both fly ash and slag.

## 1. Introduction

Nowadays, the cement industry constitutes a very contaminant sector [1,2]. For reducing its greenhouse gases emissions and improve its contribution to mitigating global warming, many strategies have been developed [3,4,5]. Among them, the use of eco-friendly cements with additions substituting clinker is becoming more and more common [6,7,8]. Most of these additions or supplementary cementitious materials are obtained from wastes generated in other industries [9,10,11,12], so the reuse of these wastes in cement manufacture would avoid other environmental impacts, also contributing to sustainability. In addition, it has been reported that the use of some of these additions produces an enhancement in the behavior of cementitious materials [10,13,14], so the study of their effects still constitutes a relevant area of investigation.

Two of the additions mostly used are fly ash and ground granulated blast-furnace slag. They belong to the group of active additions because they have hydraulic and/or pozzolanic activity [15,16]. For fly ash, it is able to react with portlandite [13,15,17] formed during the hydration reactions of clinker, giving as a result hydrated phases, which reduce the pore size, entailing a better performance of the material [10,13]. The blast-furnace slag is capable of reacting with the water since the setting of the materials, producing the formation of additional calcium-silicate-hydrate (CSH) phases, which leads to a refinement of the pore structure [10,11,12,18] and an enhancement of durability and mechanical strength [19,20].

There are also additions without the abovementioned pozzolanic or hydraulic activity that are commonly used in cement manufacture as clinker replacement. The most popular of them is limestone. This addition act as filler in cementitious materials, having a positive influence on their pore network, fresh and hardened properties [21,22,23].

With regard to the manufacture of commercial cements with additions, today, those made with binary binders are mainly produced. In these binary binders, clinker is partially replaced by one addition. However, the standards related to cement production [24] allow the possibility of substituting clinker by more than one addition in the same binder. In the case of ternary binders, in which clinker is in part substituted by two additions, their use in the production of commercial cements is still very low, particularly in Spain. In these ternary binders, incorporating two additions could bring a better performance of cementitious materials due to the possible synergetic effects of their combination [25,26,27,28]. Then, to study the behavior of cement-based materials with ternary binders [21,29,30,31] focused on the use of these binders for commercial cements manufacture would constitute a potential field of investigation for providing more alternatives to cut down the ecological impacts of cement production.

In that regard, the cement-based materials that belong to real construction elements are subject to different environmental conditions depending on their geographical location, which differ from those laboratory conditions in which their behavior is generally studied. It has been reported that the active additions are more sensitive to the variations in the hardening relative humidity and temperature because the variations in these environmental parameters could affect the development of hydration and pozzolanic reactions [32,33,34]. In this line, several authors [35,36,37,38,39,40] have studied the behavior of cement-based materials with binary binders exposed to real insitu conditions, obtaining an overall variability depending on the climate of the location in which the samples were sited. Therefore, the study of the performance of cement-based materials prepared with ternary binders exposed to real environments could be relevant for assessing if they are suitable for being used in commercial cements manufacture.

Then, the purpose of this work is to study the long-term influence produced by the exposure to a real in situ inland Mediterranean climate environment in the pore structure, mechanical performance, and durability-related parameters of mortars made with ternary binders, which incorporated the additions of limestone, fly ash and ground granulated blast-furnace slag. This exposure site verified the specifications of exposure class XC4, according to Eurocode 2 [41]. The reason for choosing the abovementioned additions was because they are mostly used in the manufacture of blended commercial cements in Spain. Furthermore, for making easier the real application of the obtained results by cement producers, the analyzed ternary binders accomplished the prescriptions for a normalized commercial cement type CEM II/B-M, defined by the Spanish and European standard UNE-EN 197-1 [24]. The performance of mortars prepared using ternary binders has been compared to that noted for mortars taken as reference, made with ordinary Portland cement without additions, as well as with other mortars prepared using binary binders, that included one of the analyzed additions as clinker substitution.

## 2. Materials and Methods

### 2.1. Materials and Sample Preparation

Mortars made with different binders were studied. The first type of mortar was the reference one, which was prepared using an ordinary Portland cement without additions CEM I 42.5 R (Spanish and European standard UNE-EN 197-1 [24]). The designation of this reference mortar was REF in the description and discussion of results.

Furthermore, mortars made with three different binary binders were also analyzed. In these binders, 30% of the cement CEM I 42.5 R was substituted by limestone, fly ash, and ground granulated blast-furnace slag, and they were, respectively, named as L, F, and S in the presentation of results.

Three ternary binders were also prepared. One of these binders included 15% slag and 15% limestone as cement CEM I 42.5 R replacement, and its designation was SL. Another ternary binder consisted of substituting the previously mentioned cement CEM I 42.5 R by 15% slag and 15% fly ash, and it was named SF. Finally, in the last of these ternary binders, whose designation was FL, the cement CEM I 42.5 R was partially replaced by 15% fly ash and 15% limestone additions. In Table 1 are shown the abovementioned designations of the binders studied and their meaning.

Limestone, ground granulated blast-furnace slag, and fly ash accomplished the specifications of the UNE-EN 197-1 standard [24] for being used as additions in the manufacture of commercial cements. The three additions studied in this research were provided by the company Cementos Portland Valderrivas (Madrid, Spain), and they are used at this time in the commercial blended cements produced by this company. The chemical compositions of these additions are gathered in Table 2.

The studied binders verified the prescriptions for a standardized commercial cement type CEM II/B, defined by the standard UNE-EN 197-1 [24]. The motivation for selecting this kind of cement is due to the fact that at present, cement type II [24] is mostly manufactured in Spain, then this may make easier a broader potential real application of the results achieved in this work.

The water to binder ratio was 0.5 for all the series of mortars. The samples were made with an aggregate to binder ratio of 3:1, and the fine aggregate used accomplished the specifications of the standard UNE-EN 196-1 [43].

Three kinds of samples were set. The first was prismatic samples with sizes 4 × 4 × 16 cm^3^. The second type of specimen was cylinders with dimensions 6 cm height and 5 cm diameter. Lastly, another kind of cylindrical sample was made, in this case with 10 cm diameter and 22 cm height.

All the samples were stored in a chamber under an optimum laboratory condition (20 °C temperature and 95% relative humidity) during the first 24 h after setting. Once ended this time, they were de-molded, and they were cured under that optimum condition up to 7 hardening days when they were moved to the real in situ exposure site. Several experimental works have shown [34,35,44] the significance of curing in the development of properties of cement-based materials exposed to non-optimum environments. For that, here has been chosen a curing period of 7 days. Finally, the tests were conducted at 28 and 250 days.

### 2.2. Environmental Exposure Condition

**Figure 1:**
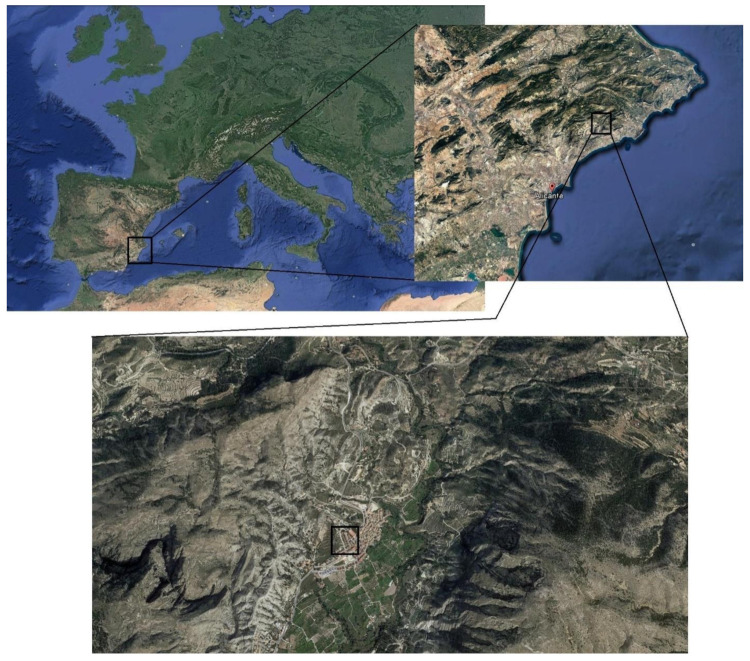
Location of the exposure site (black line squares). The specimens were placed on the roof of a detached house located in Orxeta town (Alicante province, Spain), and they were not protected from the weather conditions. The satellite images were obtained using the Google Earth software (Version 7.3.4, Mountain View, CA, USA).

The real in situ condition consisted of exposing the samples to a Mediterranean climate environment in an inland location sited in Orxeta town (38°33′47″ N, 0°15′43″ W, 177 m.a.s.l.), which belongs to Alicante province (Spain) (see Figure 1). This exposure station was not too far away from the coast (10 km approximately). The samples were placed on the roof of a detached house, and they were not protected from the weather conditions. This location would accomplish the specifications of exposure class XC4 (corrosion induced by carbonation, cyclic wet and dry) defined by the Eurocode 2 [41].

**Figure 2:**
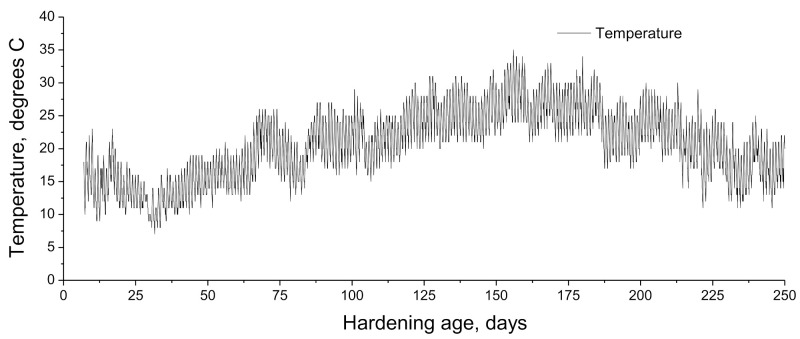
Temperature registered in the exposure station along the time period studied.

**Figure 3:**
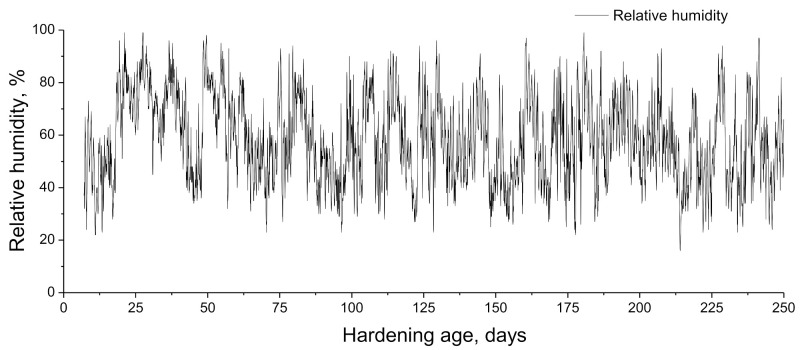
Relative humidity registered in the exposure station along the time period studied.

**Figure 4:**
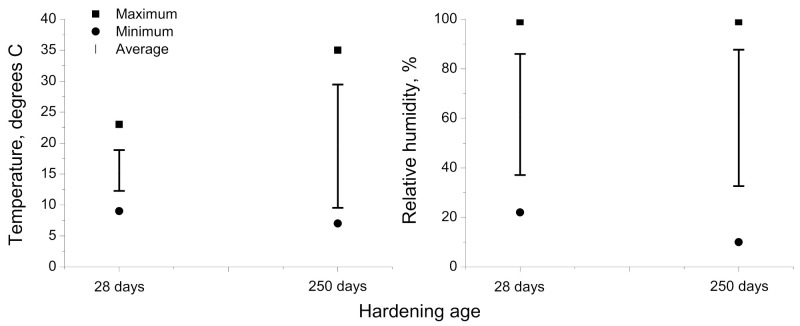
Absolute maximum, absolute minimum, and variation interval of the average daily temperature and relative humidity at which the samples tested at 28 and 250 days were exposed.

**Figure 5:**
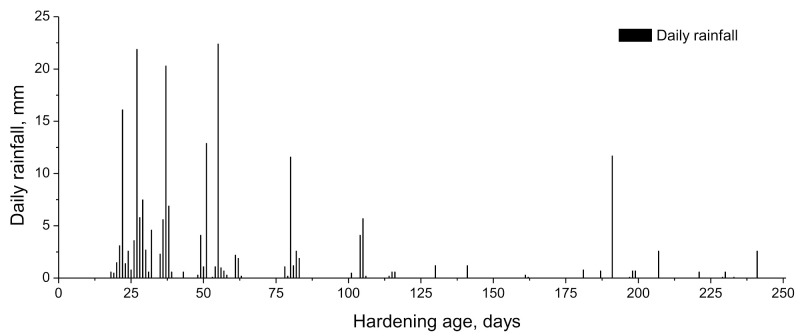
Daily rainfall registered in the station over the exposure period.

The exposure period studied in this work started at the age of 7 hardening days, once finished the curing time of the samples, as has been explained in the previous subsection, and finished at 250 hardening days, covering the months from February to October. During this period, the relative humidity and temperature in the exposure site were registered each hour. The evolution of temperature and relative humidity measured in the exposure station along the studied time interval is shown in Figure 2 and Figure 3, respectively. Moreover, in Figure 4 it is represented the absolute maximum, the absolute minimum, and the variation interval of the daily average environmental relative humidity and temperature at which the specimens tested at 28 and 250 days were exposed. On the other hand, rain is another environmental factor that may have an influence on the water saturation degree of the specimens, and as a consequence, in the development of their properties and pore network. The daily rainfall registered in the location along the exposure period is represented in Figure 5.

As can be seen, in general, both temperature and relative humidity showed high variability. With regard to the time period until 28 hardening days, it corresponded to the final of the winter season in the exposure site. The temperatures registered in that period were overall mild, ranging the daily average temperature between 12 and 18 °C, with an absolute maximum value of 23 °C (see Figure 4). In addition, along this period, it was several rainy days (see Figure 5), which produced that the environmental relative humidity registered was relatively high, particularly from 18 to 28 days (see Figure 3), with an absolute maximum value near 100% (see Figure 4).

With respect to the period between 28 and 250 hardening days, it covered the spring and the summer seasons and the initial part of the autumn. As has been expected, in this period, it has been registered higher daily average temperatures, reaching an absolute maximum value of 35 °C (see Figure 4). The relative humidity showed oscillations, alternating periods with moderately high and low values, although there were several days in which it was registered a relative humidity lower than 50% (see Figure 3), with an absolute minimum value around 10% (see Figure 4). Finally, scarce rainfall has been registered during this period (see Figure 5).

### 2.3. Mercury Intrusion Porosimetry

The mercury intrusion porosimetry technique allows getting information on the pore structure of materials [45,46,47]. In this work, the porosimeter used for performing this technique was a Poremaster-60 GT model of Quantachrome Instruments (Boynton Beach, FL, USA). The tested samples were dried in an oven at 50°C for 48 h before the porosimetry test. In this work, the total porosity and the pore size distributions determined with this technique were analyzed. For the pore size distributions, it was considered the following pore size ranges: <10 nm, 10–100 nm, 100 nm to 1μm, 1–10μm, 10 μm to 0.1 mm, and >0.1 mm [48,49]. Two measurements were made on each binder at the corresponding testing ages. Pieces taken from cylindrical specimens with 6 cm height and 5 cm diameter were tested.

### 2.4. Electrical Resistivity

The electrical resistivity allows getting information about to microstructure and connectivity of the pores in cementitious materials [50,51]. Here, the resistivity of the mortars was determined using the non-destructive Wenner four-point test, following the prescriptions of the Spanish standard UNE 83988-2 [52]. This electrical resistivity was directly measured with a Torrent permeability tester (Proceq, Schwerzenbach, Zurich, Switzerland) on cylinders with 10 cm diameter and 22 cm height several times until 250 days. Before each measurement, the surface of the cylinders was prepared according to technical recommendation number 5 of Alconpat International [53]. Three cylinders were tested for each mortar type, and four measurements were performed per specimen at each testing age.

### 2.5. Water Absorption

The absorption after immersion was obtained according to the procedure included in the ASTM Standard C642-06 [54]. Six pieces taken from cylinders with dimensions 6 cm height and 5 cm diameter were tested for each mortar type at 28 and 250 days.

### 2.6. Steady-State Chloride Diffusion Coefficient

The steady-state chloride diffusion coefficient has been obtained from the electrical resistivity of the samples saturated with water. The electrical resistivity was determined following the procedure described in Section 2.4. Specimens were water-saturated for 24 h following the standard ASTM C1202-97 [55]. Three cylinders with 10 cm diameter and 22 cm height were tested at 28 and 250 days for each binder. Four measurements were performed per specimen at both testing ages. Lastly, the steady-state diffusion coefficient was calculated with the next expression [56]:

(1) $$D_{S}=\frac{2\times 10^{-10}}{\rho }$$

where D_s_ is the chloride steady-state diffusion coefficient through the sample (m^2^/s) and ρ is the electrical resistivity of the specimen (Ω·m).

### 2.7. Carbonation Depth

The evolution of carbonation depth in the analyzed mortars was determined in accordance with RILEM (International Union of Laboratories and Experts in Construction Materials, Systems and Structures) recommendation CPC-18 [57]. Pieces obtained from the original cylindrical specimens with 6 cm height and 5 cm diameter were sprayed with a 1% phenolphthalein solution. The depth of the colorless carbonated part from the external surface of the sample was measured. For each binder, six pieces taken from the cylindrical specimens previously indicated were tested at 28 and 250 hardening days.

### 2.8. Mechanical Strengths

The compressive and flexural strengths were obtained following the prescriptions of the Spanish and European standard UNE-EN 1015-11 [58]. For each binder, three different prismatic samples with dimensions 4 × 4 × 16 cm^3^ were tested at 28 and 250 hardening days.

### 2.9. Ultrasonic Pulse Velocity

The ultrasonic pulse velocity (UPV) provides information about the mechanical performance of the material. This parameter was obtained following the procedure explained in the standard UNE-EN 12504-4 [59]. In this test, the propagation time of the ultrasonic waves in the largest dimension of the sample (160 mm) was measured with direct transmission, using a Pundit Lab model of Proceq manufacturer (Schwerzenbach, Zurich, Switzerland). Contact transducers that emitted ultrasonic pulses at 54 kHz were attached to the square sides of the samples with a coupling agent. The UPV was determined from the propagation time and the length of the sample. This parameter was obtained at several ages until 250 days. At each age, for the same binder, three prismatic specimens with dimensions 4 × 4 × 16 cm^3^ were tested, and three determinations were performed on each specimen.

## 3. Results

### 3.1. Mercury Intrusion Porosimetry

**Figure 6:**
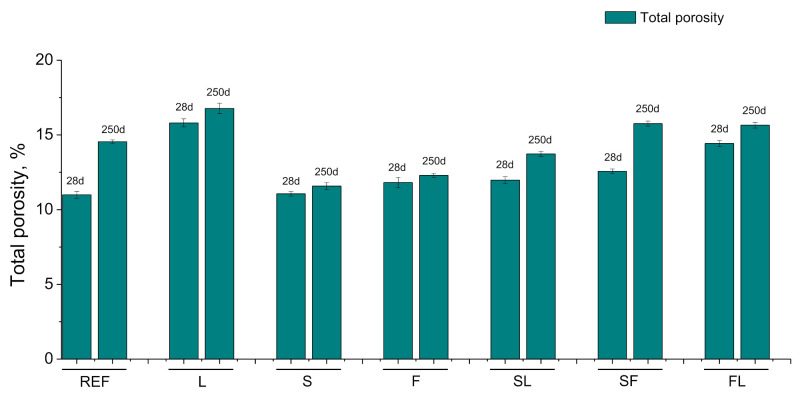
Results of total porosity for the analyzed binders. In this figure and in the next figures, the mean value of the parameter obtained for each binder is represented. In addition, the error bars represent the standard deviation.

Regarding the results of mercury intrusion porosimetry, the evolution of total porosity is shown in Figure 6. At 28 days, the smallest values of this porosity were noted for REF, S, F, SL, and SF series, with scarce differences among them. On the other hand, the highest total porosity was observed for L mortars, followed by FL ones. Between 28 and 250 days, it has been noted an increase in this parameter for all the mortars. This rise was more noticeable for the REF and SF series. At 250 days, S and F series showed the lowest total porosity values, followed by SL mortars, being higher than the porosity obtained for the REF, SF, and FL series. Lastly, the highest value of this porosity after 250 hardening days was obtained for L mortars.

**Figure 7:**
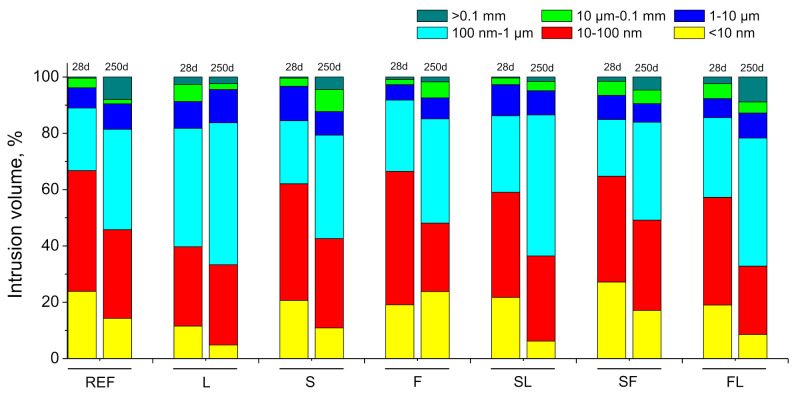
Pore size distributions obtained for the studied mortars.

In relation to the pore size distributions, they are depicted in Figure 7. After 28 days, the percentage of pores with diameters smaller than 100 nm (ranges 10–100 nm and <10 nm) was relatively similar for REF, S, F, and SF mortars, showing SF ones the highest relative volume of pores in the interval <10 nm. Regarding the other ternary binders (SL and FL series), the percentage of pores lower than 100 nm at 28 days was slightly lower compared to the abovementioned series. Between 28 and 250 days, all the analyzed binders showed a noticeable reduction in the percentage of finer pores. At the last age studied, the relative volume of pores with diameters lower than 100 nm was greater for F and SF series in comparison with other binders. At 28 and 250 days ages studied, the lowest percentage of finer pores was observed for L mortars.

### 3.2. Electrical Resistivity

**Figure 8:**
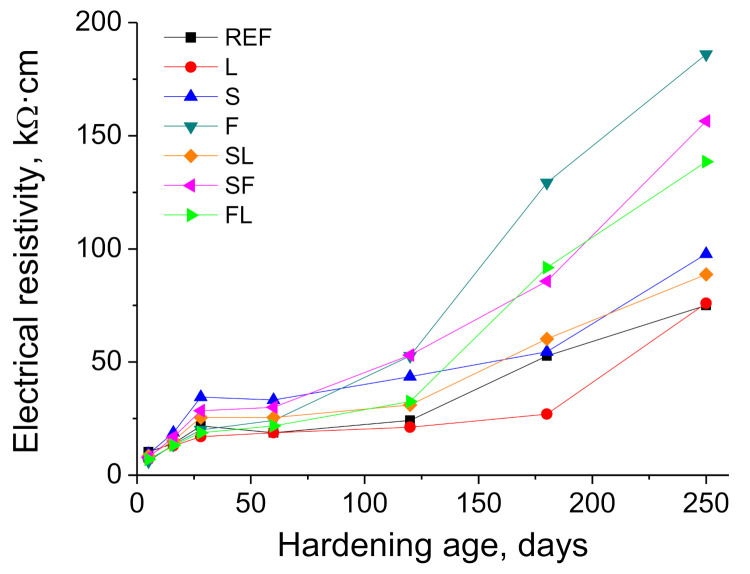
Electrical resistivity results noted for the analyzed mortars.

The electrical resistivity results can be observed in Figure 8. In the short term, the binary and ternary binders that incorporated slag (S, SL, and SF series) showed higher values of this parameter, particularly S mortars. Since 60 days approximately, mortars made with the binary binder with fly ash (F series) started a noticeable growth of their electrical resistivity, presenting the highest values of this parameter at 250 days. For the ternary mortars with both limestone and fly ash additions (FL series), the tendency was similar, although the more notable increase in electrical resistivity started later. In the long term, the greatest values of the resistivity were noted for mortars with fly ash (F series followed by FL and SF binders), being lower for the other mortars with slag (S and SL series). Finally, REF and L mortars overall showed the lowest electrical resistivity at later ages.

### 3.3. Water Absorption

**Figure 9:**
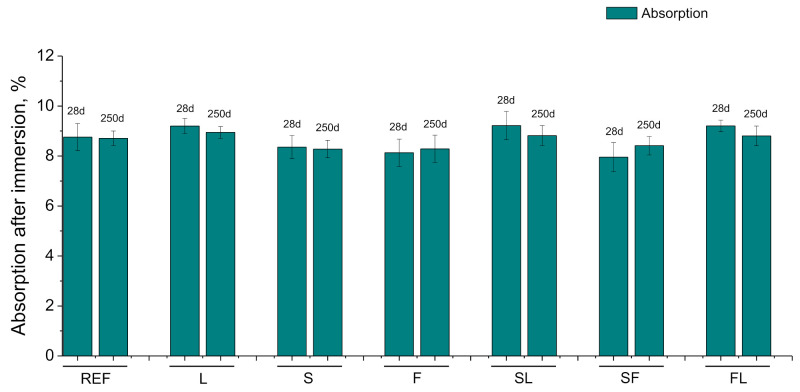
Results of absorption after immersion for the studied binders.

The results of water absorption after immersion are represented in Figure 9. This absorption was slightly lower for S, F, and SF series in comparison with the other analyzed binders. In general, small reductions inthe time of absorption after immersion have been noted for all the studied binders. After 250 days, it was scarce higher for binary and ternary binders with limestone addition (L, SL, and FL series).

### 3.4. Steady-State Chloride Diffusion Coefficient

**Figure 10:**
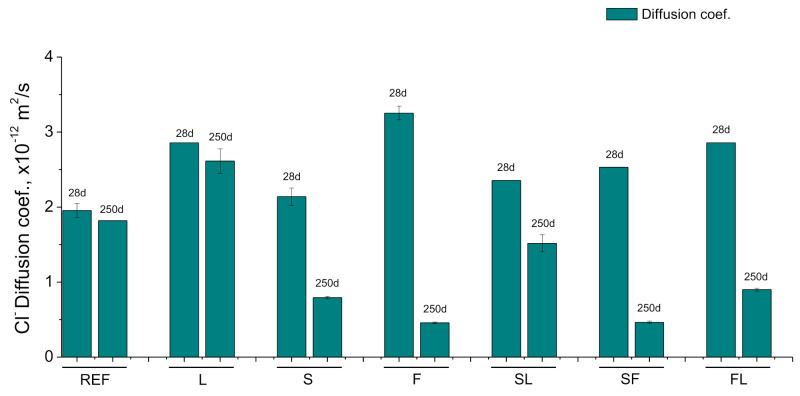
Results of steady-state chloride diffusion coefficient obtained from resistivity of water-saturated samples.

The results of the steady-state chloride diffusion coefficient obtained from the saturated sample’s resistivity are shown in Figure 10. At 28 days, this coefficient was lower for REF mortars and for specimens prepared with binary and ternary binders with slag (S, SL, and SF series), compared to the other binders with fly ash and limestone (F, FL, and L mortars). Between 28 and 250 days, the diffusion coefficient showed a decrease for mortars that incorporate fly ash and slag (S, F, SL, SF, and FL series), being more noticeable the reduction in this parameter for specimens with fly ash in the binder (F, SF and FL series). For REF and L mortars, the diffusion coefficient hardly decreased between 28 and 250 days. At that last age studied, the smallest coefficients were noted for SF and F specimens, nearly followed by S and FL ones. On the other hand, the diffusion coefficient was scarce greater for SL and REF series at 250 days in comparison with the abovementioned series, while the greatest value of this coefficient at that age was noted for the L series.

### 3.5. Carbonation Front Depth

**Figure 11:**
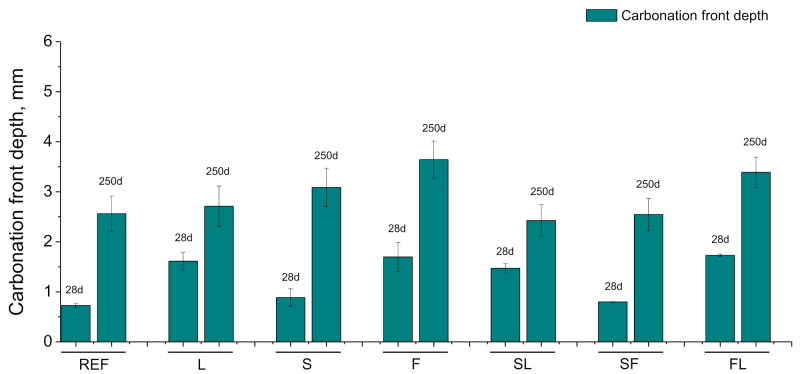
Carbonation front depths noted for the different binders studied.

The results of carbonation front depth measurements can be observed in Figure 11. In general, there were no excessive differences in this parameter comparing the different mortars tested. At 28 days, the carbonation depths were lower for REF, S, and SF mortars, with relatively similar values between them. On the other hand, the depths were also similar at that age for L, F, SL, and FL mortars, although they showed higher values compared to the abovementioned series. From 28 to 250 days, the carbonation front depth rose for all the analyzed series. At 250 days, the highest depths were observed for F and FL mortars. On the other hand, ternary binders with slag (SL and SF) showed at that age very similar carbonation depths to REF specimens.

### 3.6. Mechanical Strengths

**Figure 12:**
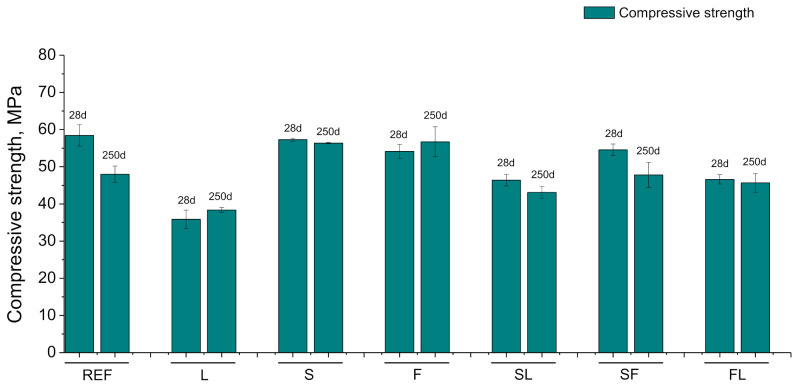
Compressive strength results for the studied binders.

The results of compressive strength are represented in Figure 12. At the age of 28 days, the highest values of this strength corresponded to REF and S specimens, followed by F and SF ones. Between 28 and 250 days, this parameter decreased for REF, SL, and SF series, being more noticeable this reduction for REF mortars, and it kept practically constant for the other binders studied. In the long term, S and F mortars showed higher compressive strength than REF. For the analyzed ternary binders, at 250 days, the compressive strength of the SF series was very similar to that noted for REF mortars, while it was slightly lower for SL and FL series compared to those reference specimens. Finally, the lowest compressive strength was obtained for L mortars at 28 and 250 days.

**Figure 13:**
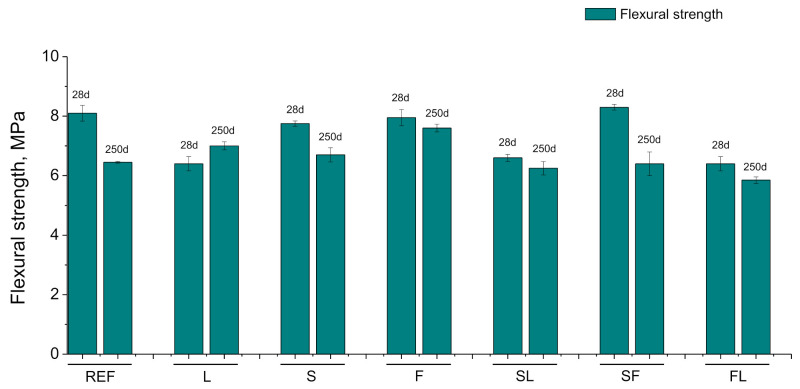
Results of flexural strength noted for the analyzed mortars.

The flexural strength results are depicted in Figure 13. At 28 days, this parameter was relatively similar for the REF, S, F, and SF series, being higher than the other binders studied. Generally, it was observed a decrease in flexural strength with time for the majority of the mortars. The reduction in this parameter was more notable for REF, S, and SF specimens. At 250 days, scarce differences in flexural strength have been noted between the different analyzed binders, being the values of this parameter in the range from 6 to 7 MPa approximately for most of them.

### 3.7. Ultrasonic Pulse Velocity

**Figure 14:**
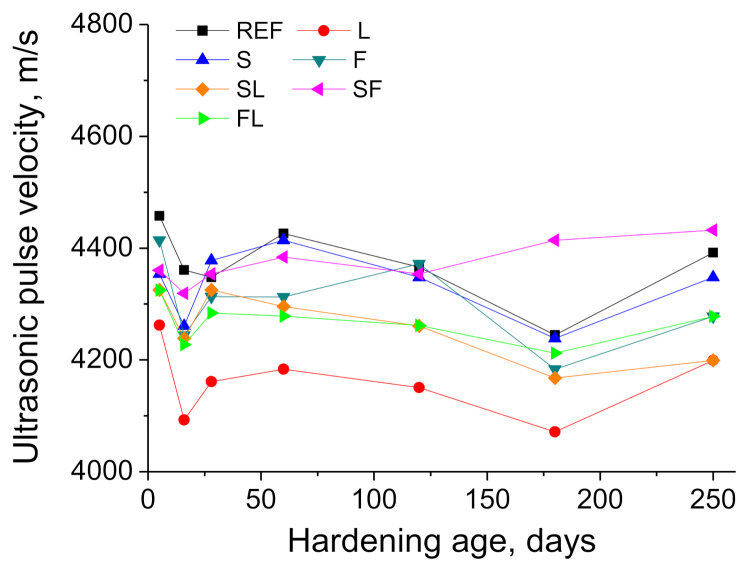
Ultrasonic pulse velocity results observed for the different types of mortars tested.

The results of ultrasonic pulse velocity (UPV) are shown in Figure 14. At initial ages, this parameter was higher for the REF, F, SF, and S series compared to the other studied binders. In general, this parameter hardly increased or even slightly decreased with time for most of the binders. Along the studied time period, UPV values noted for the majority of the series were relatively similar, being in the range from 4200 to 4400 m/s approximately. Lastly, the lowest UPV values have been overall noted for L mortars during the analyzed period of time.

## 4. Discussion

### 4.1. Microstructure Characterization

With respect to the results of mercury intrusion porosimetry technique (see Figure 6 and Figure 7), the very similar total porosity and the higher pore refinement, with a greater presence of finer pores, noted at 28 days for reference mortars and some of the binary and ternary binders with slag and fly ash (S, F and SF series), would be linked to the development of clinker and slag hydration [19,60] and fly ash pozzolanic reactions [13]. These reactions produced solid phases that progressively closed the microstructure, reducing the porosity of the material and increasing the percentage of pores with smaller diameters [10,18].

For initiating the development of fly ash pozzolanic reactions, it is needed the presence of enough portlandite produced by clinker hydration, so the effects of these pozzolanic reactions on the microstructure are generally delayed, compared to effects of slag and clinker hydration [13]. At 28 days, scarce differences in total porosity and pore size distributions were noted between the binary binder with fly ash (F series) and reference mortars (REF series) and those with only slag as an addition (S series). This would suggest that it has been produced noticeable progress in the short term of fly ash pozzolanic reactions. This result could be related to the environmental conditions in the exposure site up to 28 hardening days. In this period, it rained all days from 18 to 28 days, and in some of them, it was noted that daily rainfalls were higher than 15 mm (see Figure 5). In addition, in that period, the environmental relative humidity registered was relatively high (see Figure 3), reaching maximum values of this parameter around 100% (see Figure 4). The combination of the high relative humidity and several rainy days in that period would produce a rise of water content in the specimens, which even probably were saturated with water in some time periods along the first 28 hardening days. The greater availability of water supplied by the environment would facilitate the development of hydration reactions of clinker components [33,60], so a higher presence of portlandite would be available at short ages. This would allow a sooner initiation of fly ash pozzolanic reactions and their greater development in the short term, as suggested by the mercury intrusion porosimetry results.

On the other hand, the temperature of the environment also has an influence on the development of the hydration and pozzolanic reactions [32,33,60,61,62,63]. In this line, it has been reported that low temperatures would slow down these abovementioned reactions [32,33,60,61,62,63]. Up to 28 hardening days, the temperatures registered in the exposure site were mild (see Figure 2), reaching a maximum value of 23°C and ranging the daily average temperature from 12 to 18°C (see Figure 4). Therefore, it would be expected that these temperatures in the exposure site would scarcely slow down the development of hydration and pozzolanic reactions [32,60,61,63]. These environmental conditions up to 28 hardening days (mild temperatures, high relative humidity, and several rainy days) would also have a beneficial effect on the slag hydration [60,62,63], as suggested the similar pore size distribution and total porosity at that hardening time observed for S mortars compared to REF ones because a relatively high presence of water provided by the environment would facilitate the slag hydration [33].

Regarding the analyzed ternary binders, the most refined pore structure at 28 hardenings was noted for the SF series, which combined both slag and fly ash additions. This could be due to the synergetic effects of the hydration of slag and pozzolanic reactions of fly ash [64], considering the previously discussed influence of the environment in the microstructure development, giving, as a result, a higher percentage of pores in the interval <10 nm in this binder at that age compared to REF, S and F mortars, with similar total porosity. With respect to the ternary binders with limestone (SL and FL mortars), a slight loss of pore refinement at 28 days has been noted in comparison with the binary binders with slag and fly ash additions (S and F series). This may be related to the presence of limestone, which is an addition without hydraulic or pozzolanic activity [21,65], only having a filler effect in the microstructure, so its incorporation would not produce an additional solid phases formation, as occurred with fly ash and slag. Despite that, the development of slag hydration and fly ash pozzolanic reactions, favored by the high relative humidity and water provided by the environment, as has been previously explained, would reduce the influence of limestone in the microstructure of these ternary binders at 28 days. Finally, the less pore refinement and higher porosity noted for L mortars at that age would be due to the abovementioned effects of this not active addition, which would act as an inert filler material without reactivity [65].

From 28 to 250 days, it has been registered a rise of total porosity and a pore refinement loss for all the mortars studied. This may be related to the environmental conditions because part of that time period covered the summer season in the exposure site, in which it has been registered higher average temperatures (see Figure 4), a higher number of sunshine hours, lower relative humidity (see Figure 4) and very little rainfall (see Figure 5). These conditions would produce drying of the materials, giving as a result that less water was available for the development of hydration and pozzolanic reactions at later times [60,61], as well as the possible formation of shrinkage microcracking [60,66,67]. This would entail a rise of total porosity, also an increase in the proportion of pores with higher sizes, as revealed by the porosimetry results at 250 days. In addition, exposure to an outdoor environment may produce the development of carbonation, as can be observed in Figure 11. Several authors [68,69,70] have pointed out that in the long term, the carbonation process would lead to a reduction in porosity, particularly when ordinary Portland cement without additions is used, and to a reduction in pore refinement. Then, the rise of porosity with time noted for all the studied mortars would suggest that the drying shrinkage would be the harmful process prevailing in the exposure site at later ages.

Comparing the pore size distributions obtained at 250 days between the analyzed mortars, they overall agree with those noted at 28 days, already discussed. The pore structure was more refined for the F, S, SF, and REF series, showing the abovementioned effects of hydration and pozzolanic reactions [10,13,60], as well as the synergistic effects of introducing fly ash and slag in the same binder [64]. On the contrary, mortars with limestone (L, SL, and FL series) showed less pore refinement, probably due to the fact that this addition does not have hydraulic or pozzolanic activity [65], as has been previously explained. Furthermore, several works [32,36,71] have revealed that a relatively high hardening temperature may induce faster slag hydration, which produces the formation of dense hydrated phases around the particles without reacting, avoiding later hydration. This non-uniform distribution of the hydration products could lead to a less refined microstructure in the long term, with the presence of larger pores in the pore network [32,36,71]. This could also contribute to the relatively high coarsening of the pore structure between 28 and 250 days noted for binders with only slag as active addition (S and SL series), in combination with the effects of drying shrinkage and carbonation harmful processes.

On the other hand, when the mercury intrusion porosimetry results obtained in the long term for the real condition studied in this research are compared to the results noted in a previous work of the authors [42], in which mortars with the same compositions were exposed to an optimum laboratory condition, it is important to highlight the higher total porosities and lower microstructure refinement observed for all the mortars hardened under the real environment. As has been previously explained when it has been discussed the evolution of the pore network from 28 to 250 days, the environmental conditions in the real exposure site produced in the long term a progressive drying of the material [60,61], making more difficult the development of slag and clinker hydration, as well as the fly ash pozzolanic reactions, which need the presence of enough water for their progress, also leading to the possible formation of shrinkage microcracking. This would not happen under an optimum laboratory condition, where there was enough water available for developing those reactions, and no drying process was produced. This would mainly explain the microstructural differences in the studied mortars between the real and optimum conditions. Furthermore, the carbonation process produced in the real condition could have also contributed to those differences.

The electrical resistivity allows obtaining information regarding the changes in the pore structure of cement-based materials, although it is greatly influenced by the drying process, which would modify the saturation degree of the material [53,72]. This drying produces a lessening of the volume of electrolyte, which fills the pore structure of the material, giving, as a result, an increase in the electrical resistivity [72,73,74]. The reduction in porosity and the greater presence of pores with finer sizes would also lead to a reduction in the amount of electrolyte present in the material, also producing a rise of the resistivity [75,76,77]. Here has been noted a progressive increase with time of this parameter for all the series tested (see Figure 8). In the short term, until 28 days approximately, the rising tendency of the electrical resistivity would be more related to the microstructure closing, as a consequence of the development of hydration and pozzolanic reactions, facilitated by the high environmental relative humidity during that time period (see Figure 3 and Figure 4), as has been explained for porosimetry results. However, since then, this parameter has increased with a progressively higher rate, in particular during the summer season. The pore size distributions noted at 250 days showed a lessening of pore structure refinement, so the long-term increase in resistivity may be more related to the drying of the material, which would be compatible with the conditions of the exposure site in the warmer months (higher temperature, lower relative humidity, higher number of daylight hours and scarce rainfall). In addition, comparing the electrical resistivity results noted in this work for the analyzed real environment and those obtained for mortars with the same composition hardened in an optimum laboratory condition [42], the values of this parameter were generally higher for the real environment, whereas their microstructure was less refined than in the optimum condition. This would corroborate the abovementioned effect of the drying process in the resistivity evolution for mortars exposed to the real environment.

Despite that, another factor that could also have an influence on the differences between the porosimetry and electrical resistivity results at later ages would be the different types of samples used for both techniques. For mercury intrusion porosimetry, the tested pieces were extracted from small cylinders (5 cm diameter and 6 cm height). Nevertheless, the electrical resistivity was measured in another kind of cylindrical samples, with greater volume and dimensions (10 cm diameter and 22 cm height). The smaller size of the cylinders used for obtaining the porosimetry samples would entail a faster drying of the entire sample, showing more uniform effects of the environment in the microstructure. In the case of the larger cylinders in which the electrical resistivity has been determined, the effects of the environment, especially the drying process, in the internal part of the sample would be delayed, keeping during more time enough electrolyte available in the microstructure for developing the hydration and pozzolanic reactions, resulting in a higher microstructure refinement in that internal part of the cylinders. The electrical resistivity measurements permit to get more global information about the pores of the sample, so this parameter may be influenced by the possible different microstructure development between the internal and external parts of the sample. This could contribute to explain the different results between porosimetry and electrical resistivity, together with the abovementioned influence of the drying in this parameter.

In relation to the resistivity noted at later ages for the analyzed binders, with a similar drying degree produced by the exposure environment, several coincidences with the previously discussed results have been observed. Firstly, mortars with active additions showed higher electrical resistivity, being in accordance with the results pointed out by other authors [72]. The greater values at 250 days of this parameter noted for F and SF mortars, in comparison with the rest of the binders, would agree with their higher percentage of smaller pores at that age. Moreover, the lower resistivity values for fly ash binders in the short term, compared to specimens with slag, and their progressive growth with time, even exceeding in the long term the values noted for slag binders, would suggest the delay of the pozzolanic reactions of fly ash respect to the hydration of slag [13,17], already explained. Finally, the limestone addition produced a reduction in electrical resistivity when it was incorporated into the binder, as showed the comparison between mortars with slag (SL series had lower resistivity compared to S and SF series) and between those with fly ash (FL series had lower resistivity in comparison with SF and F series). This would be in accordance with pore size distributions, being in relation to the only filler effect of limestone [21,65], without hydraulic or pozzolanic activity, previously discussed.

### 4.2. Durability and Mechanical Parameters

With respect to the durability of the studied binders, the overall reduction with time of the steady-state chloride diffusion coefficient (see Figure 10) would agree with the increase in electrical resistivity, suggesting a pore refinement with time, which would not be in line with the evolution of pore size distributions. The diffusion coefficient has been calculated from the electrical resistivity of water-saturated cylinders with 22 cm height and 10 cm diameter (see Section 2.6), analogous to those used for studying the resistivity in the non-saturated material. Among the arguments previously explained for justifying the electrical resistivity results, the influence of the saturation degree in the measurements would not be applicable for the diffusion coefficient because the samples were saturated in the water previously to register their electrical resistivity. Therefore, the differences between the diffusion coefficient results and the pore size distributions would be due to the possible higher microstructure refinement in the internal part of the samples, compared to their external part, discussed with detail for the electrical resistivity results in the previous subsection.

At 28 days, the diffusion coefficient results showed similarities with those obtained in the microstructure characterization. The lower coefficients noted for reference mortars and those with slag (REF, S, SF, and SL series), compared to other series with fly ash (F and FL mortars), would show once again the delay the pozzolanic reactions of fly ash with respect to the hydration of clinker and slag [13,17], although this delay seemed to be more noticeable in the diffusion coefficient than in the pore size distributions. The slight decrease with time of this coefficient observed for REF and L mortars would suggest that the drying effect of the environment would affect the internal part of their samples earlier than in the specimens with active additions (slag and/or fly ash), probably as a consequence of a less refined microstructure. On the other hand, the environmental influence in the core part of the sample would be lower in the long term for those series with active additions, as would indicate their notable decrease with time of diffusion coefficient. This would be in keeping with their greater refinement of pores, which would make more difficult the drying of the microstructure electrolyte, thus delaying the harmful effects of the environment. In this line, it is interesting to highlight that S, F, SL, SF, and FL series showed lower diffusion coefficients than reference specimens. With regard to the comparison of the long-term results of this parameter between the real environment studied in this research and those previously published for the same materials under an optimum condition [42], it has been observed higher diffusion coefficient values in the real environment for all the mortars. This would agree with the differences of mercury intrusion porosimetry results between both conditions, already discussed. However, it is interesting to highlight that these differences were lower for this coefficient, compared to other parameters analyzed, which would be in keeping with the possible less influence of the real environment in the core part of the sample, at least until the latest studied age of 250 days.

The lower values of the water after absorption after immersion noted for S, F, and SF series compared to the other studied mortars would be consistent with the other parameters described. Nevertheless, the small changes with time and the overall slight differences between the analyzed mortars regarding the water absorption after immersion (see Figure 9) would not totally agree with the results previously discussed. The relatively similar values of the water absorption, independently of the binder, may be influenced by the method used for obtaining this parameter, defined in the ASTM Standard C642-06 [54]. The initial phase of this method required keeping the specimens at 100 to 110°C at least 24 h before they were saturated [54]. This drying at high temperature could produce the development of shrinkage cracks in the mortars [16,67,78], which would cover up and notably remove the influence of the environment, entailing a more uniform performance of the mortars in relation to the water absorption parameter.

The development of carbonation with time in the samples (see Figure 11) would be expected due to their exposure to a real outdoor environmental condition, in contrast with the lack of carbonation of the same materials hardened under an optimum laboratory condition that was studied in previous work [42]. The differences regarding the carbonation front depths between the analyzed binders in this real environment were not very high. In general, these depths were greater during the analyzed period of time for F and FL mortars, which could be explained in relation to the consumption of portlandite during the fly ash pozzolanic reactions, as has been reported by several authors [16,69]. Moreover, the carbonation process would produce a reduction in pore refinement due to the formation of silica as a product of the decomposition of C-S-H gel caused by the CO_2_ exposure [15,16,70]. Then, the rise with time of carbonation depths would agree with the results of pore size distributions, showing that carbonation could also contribute to the loss of microstructure refinement, in addition to the already explained effects of drying shrinkage.

The decreasing tendency with time of the mechanical strengths (see Figure 12 and Figure 13) for most of the studied binders would be in consonance with the reduction in porosity and the coarsening of pore structure discussed before. This may be related to the conditions of the exposure site, mainly with the drying of the materials in the long term [34,67,79], although the carbonation could also have helped to the reduction in mechanical performance because this process has harmful effects in these properties, according to several authors [80,81]. It was noticeable that the relatively high compressive and flexural strength at 28 days was noted for the F series, very similar to reference mortars and binary binder with slag (S series). This result would also be consistent with those obtained with mercury intrusion porosimetry, pointing out a possible moderate development of fly ash pozzolanic reactions, probably favored by the high relative humidity and the heavy rain registered in the exposure station at short hardening ages, as has been explained. In addition, the incorporation of limestone in ternary binders (FL and SL series) generally reduced the mechanical performance of the mortars, compared to the rest of binders with slag and/or fly ash (S, F, and SF series). These results were also coincident with most of those already described, showing the effects of this non-active addition [21,65]. Lastly, it is interesting to highlight the similar or even better flexural and compressive strengths observed at 250 days for most of the binders with at least one active addition (slag and/or fly ash) in comparison with reference mortars.

The ultrasonic pulse velocity (UPV) results were overall in keeping with those obtained for mechanical strengths. The higher UPV values noted at early ages for REF, F, SF, and S mortars (see Figure 14) would agree with their slightly higher compressive and flexural strengths at 28 days, showing the effects of hydration and pozzolanic reactions [10,13,60], favored by the mild environmental conditions in the short term, as has been discussed in the microstructure characterization. The constant or decreasing trend of the UPV with time for most of the binders studied would be in consonance with the general reduction in the mechanical strengths from 28 to 250 days, produced by the explained influence of the environment. The smallest UPV registered for the L series along the analyzed exposure period coincided with their lowest compressive strengths, corroborating the abovementioned influence of the limestone addition in the mechanical behavior of the mortars under the studied environment.

Finally, if the mechanical performance results obtained in the real environment studied in this research are compared to those noted in a previous work of the authors [42], in which the influence of an optimum condition in mortars with the same composition was studied, it is interesting to point out that in the long term the mechanical strengths and the ultrasonic pulse velocity values were generally lower in the real environment than in the optimum condition for all the analyzed mortars. This would be in agreement with the results of comparing both environments for the other parameters studied, which were related to the microstructure characterization and durability-related properties. According to the discussion of those results, already explained, the high relative humidity available in the optimum environment would allow adequate development of hydration and pozzolanic reactions [13,17,33,42], improving the mechanical performance of the mortars. On the other hand, the harmful processes developed in the real environment, such as the drying shrinkage and the carbonation process, would produce a worsening of the mechanical performance of the materials [34,67,79] compared to the optimum condition, as has been observed here.

## 5. Conclusions

The main conclusions that can be obtained from the results previously discussed can be summarized as follows:

- The relative mild and humid environmental conditions during the first weeks of the exposure period improved the microstructure and the properties of the studied mortars, facilitating the development of clinker and slag hydration, as well as the pozzolanic reactions of fly ash at early ages;
- The studied binders generally showed an increase in total porosity, a loss of pore refinement, a rise of the carbonation front depths, and a reduction in their mechanical strengths with time. This could be due to the long-term effects of the warm environmental conditions in the exposure site, which would produce a progressive drying of the material, making more difficult the development of hydration and pozzolanic reactions and probably producing the formation shrinkage microcracking. This would give, as a result, the worsening with the time of the performance of the mortars, to which the development of carbonation would also contribute, although to a lesser extent. In addition, these harmful effects would also lead to the worst long-term behavior of the mortars exposed to the studied real environment, compared to their performance under an optimum laboratory condition pointed out in previous work;
- The electrical resistivity showed a progressive rise with age for all the studied binders, which would be due to the reduction in the amount of the electrolyte that fills the microstructure of the material, mainly produced by the drying process as a consequence of the exposure to the real environment. However, this increase with time of the resistivity could also be related to a different microstructure development in the internal and external parts of the samples used for measuring this parameter due to their higher size and volume than those tested with the other techniques. This would produce a denser microstructure of the material in the samples’ core part, influencing the resistivity. In addition, the reduction with the age of the steady-state chloride diffusion coefficient observed would be in keeping with the increase in electrical resistivity, which would also point out the abovementioned possible differences between the internal and external parts of the specimens;
- The results of the water absorption after immersion would be affected by the drying in an oven at 100°C established in the experimental procedure performed, masking and notably removing the influence of the environmental conditions in this parameter. Despite that, the binary binders with slag and fly ash (S and F series) and the ternary binder, which incorporated both additions (SF series), showed lower water absorption values than other analyzed mortars;
- The differences in relation to the carbonation front depths between the binders studied were not high, although in general, they were greater for binders with only fly ash as active addition (FL and F series). This result may be due to their lower portlandite content as a consequence of the development of the pozzolanic reactions of fly ash;
- After 250 days, the binary binders with slag and fly ash (S and F mortars) and the ternary binder, which incorporated both additions (SF series) overall, showed higher pore refinement and similar or even better mechanical performance than reference mortars without additions. This would be due to the effects of the hydration of slag and the pozzolanic reactions of fly ash, as well as by the synergetic effect of combining both additions in the ternary binder;
- The incorporation of limestone in the ternary binders (FL and SL mortars) would entail a lower pore structure refinement and a reduction in the mechanical strengths, compared to the other binders with slag and/or fly ash (S, F, and SF series). This may be explained in relation to the lack of hydraulic or pozzolanic activity of limestone addition, so its effects are limited in comparison with slag and fly ash, acting only as a filler material.

## Figures and Tables

**Table 1:** Designation of the mortars studied and percentage (in weight) of CEM I and additions [42].

Designation	CEM I 42.5 R	Limestone	Blast-Furnace Slag	Fly Ash
REF	100%	-	-	-
L	70%	30%	-	-
S	70%	-	30%	-
F	70%	-	-	30%
SL	70%	15%	15%	-
SF	70%	-	15%	15%
FL	70%	15%	-	15%

**Table 2:** Chemical components of limestone, fly ash, and blast-furnace slag.

Components	Blast-Furnace Slag	Fly Ash	Limestone
MgO	6.98%	1.40%	0.47%
Al_2_O_3_	10.10%	27.70%	1.22%
SiO_2_	31.50%	54.40%	2.85%
SO_3_	1.94%	0.53%	0.10%
K_2_O	0.52%	3.12%	0.18%
CaO	46.80%	2.55%	94.40%
TiO_2_	0.94%	1.05%	0.11%
MnO	0.17%	0.06%	-
Fe_2_O_3_	0.37%	8.06%	0.54%
P_2_O_5_	0.02%	0.46%	0.02%
Na_2_O	0.30%	-	-
ZnO	-	0.11%	-

## Data Availability

The data that support the findings of this study are available from the corresponding author, J.M.O., upon reasonable request.
